# Soil-Borne Microbial Functional Structure across Different Land Uses

**DOI:** 10.1155/2014/216071

**Published:** 2014-08-10

**Authors:** Eiko E. Kuramae, Jizhong Z. Zhou, George A. Kowalchuk, Johannes A. van Veen

**Affiliations:** ^1^Department of Microbial Ecology, Netherlands Institute of Ecology (NIOO-KNAW), 6708 PB Wageningen, The Netherlands; ^2^Institute for Environmental Genomics, University of Oklahoma, Norman, OK 73019, USA; ^3^Department of Biology, Utrecht University, 3512 JE Utrecht, The Netherlands; ^4^Institute of Biology, Leiden University, 2311 EZ Leiden, The Netherlands

## Abstract

Land use change alters the structure and composition of microbial communities. However, the links between environmental factors and microbial functions are not well understood. Here we interrogated the functional structure of soil microbial communities across different land uses. In a multivariate regression tree analysis of soil physicochemical properties and genes detected by functional microarrays, the main factor that explained the different microbial community functional structures was C : N ratio. C : N ratio showed a significant positive correlation with clay and soil pH. Fields with low C : N ratio had an overrepresentation of genes for carbon degradation, carbon fixation, metal reductase, and organic remediation categories, while fields with high C : N ratio had an overrepresentation of genes encoding dissimilatory sulfate reductase, methane oxidation, nitrification, and nitrogen fixation. The most abundant genes related to carbon degradation comprised bacterial and fungal cellulases; bacterial and fungal chitinases; fungal laccases; and bacterial, fungal, and oomycete polygalacturonases. The high number of genes related to organic remediation was probably driven by high phosphate content, while the high number of genes for nitrification was probably explained by high total nitrogen content. The functional gene diversity found in different soils did not group the sites accordingly to land management. Rather, the soil factors, C : N ratio, phosphate, and total N, were the main factors driving the differences in functional genes across the fields examined.

## 1. Introduction

Nutrient cycling within terrestrial ecosystems is mostly performed via the activities of soil-borne microorganisms [1]. With the advent of molecular biological methods, considerable amount of knowledge has been accumulated, concerning the diversity and distribution of microorganisms in soil environments [2–4]. Most of the studies related to the impact of land use change on microbes have focused on the phylogenetic composition of the soil microbial community. With respect to microbial functions in soils, most studies have traditionally been based on enzyme activity screening, with relatively little attention paid to functional marker gene screening [5]. The use of functional gene markers to monitor the presence and activity of genes responsible for key steps in terrestrial nutrient cycles may provide a much more directed approach to the analysis of the nutrient cycling properties of terrestrial ecosystems.

A large amount of knowledge is becoming available concerning the microbial enzymes responsible for the key steps of the major nutrient cycles in soil (i.e., carbon, nitrogen, sulphur, etc.). Recent studies have revealed a great diversity within the genes encoding these key enzymatic processes [6–10], providing an expanding database representing the known diversity of genes encoding key enzymatic steps involved in nutrient cycling. Microarray technologies have made it possible to represent the diversity of key enzyme functions as an array of probes, which can be interrogated with DNA or RNA extracted from the environment [11–13]. In this way, the total metagenome of an environmental sample can be examined for the presence, diversity, and activity of genes critical to the major nutrient cycles. Coupling such data with nutrient flux measurements, enzyme activities, and other measures of soil quality (including phylogenetic microarray data) could potentially provide a quantum advance in our understanding of nutrient cycling in soil systems [14]. Although sequence databases are becoming rather extensive, we clearly have yet to detect the full expanse of the diversity of key enzyme functions, and such microarray-based analyses still necessarily fail to cover all gene families that may be critical to nutrient cycling. Thus, as our knowledge of gene diversity increases, so too will our ability to design probes to monitor a broader range of genes and activities, and such functional microarrays will continue to improve and become more complete as research progresses.

Anthropogenic perturbations (e.g., pollution, fertilizer deposition, and habitat destruction) are known to influence soil nutrient cycles, but little is known about the mechanistic aspects of such disturbances. This lack of knowledge inhibits our ability to assess the extent to which human activities disturb terrestrial nutrient cycling potential and thwarts efforts to predict future anthropogenic impacts. Before the influence of such perturbations can be established or predicted, one must characterize the natural variation and normal operating range with respect to the diversity and expression of key genes related to nutrient cycle functions. To establish such normal operating boundaries, the dynamics of the gene diversity and expression must be monitored across relevant spatial and temporal scales and in response to natural and imposed variability. Although this still remains technically challenging, with hurdles related to limits of signal detection and reproducibility [15, 16], functional microarray platforms provide a powerful, high throughput, tool for the detailed assessment of microbial nutrient cycling activities.

In the current study, we exploited the functional structure of microbial communities in soils under different land uses. First, we determined the soil parameters of each field. Next, we tracked the microbial functional communities across land uses. Lastly, we identified links between soil parameters and microbial functions. The main questions addressed in this study were how does the microbial community functional structure vary with different soil managements and what are the main drivers that are related to this variation. In order to answer these questions, we used functional gene arrays (FGA), focussing on crucial steps of key nutrient cycles (C, N, P, and S) across eight fields representing five generally representative forms of land use (conventional arable field, organic arable field, pasture, natural grassland, and pine forest) in The Netherlands. To our knowledge, this is one of few studies to apply functional microarray technique across a range of different land managements. In addition, this study is one of the first to include a suite of soil measurements to explore the environmental factors driving soil microbial functions under different land uses.

## 2. Material and Methods

### 2.1. Experimental Design, Sampling, and Soil Analyses

Eight fields subjected to five generally representative forms of land management in The Netherlands (pine forest, natural grasslands, pasture, conventional arable field, and organic arable field) were sampled (Figure S1, Supplementary Material available online at http://dx.doi.org/10.1155/2014/216071) in May 2007; see Table 1. The eight fields were selected from a previous study [17] on soil factors driving microbial community composition in 26 fields across The Netherlands under different land management. In each field, a central point was selected, and subsequently four sampling points at 20 m of the central point were chosen so as to obtain five samples per field (A, B, C, D, and E). Each sample (A, B, C, D, and E) was comprised of five subsamples (A1, A2, A3, A4, A5; B1, B2, B3, B4, B5, etc) from soil cores (8 cm diameter × 20 cm deep) taken randomly within a two-meter radius of each of the five sample points A, B, C, D, and E. Soil samples were sieved through a 4 mm mesh to remove stones, roots, and plant materials. Equal amounts of each of the five subsamples of a given sampling point were pooled, thereby yielding a replication of five composite samples per field. Each composite sample was divided into two parts. One part was stored at −80°C for DNA extraction and the other part kept at 4°C for physical and chemical analysis. For physical and chemical analysis, equal amounts of each of the five replicates per field were pooled.

Physicochemical characterization was performed by BLGG (Bedrijfslaboratorium voor Grond en Gewasonderzoek, Wageningen, The Netherlands, http://blgg.agroxpertus.nl/). Soil pH was measured in a 1 : 2.5 soil/water suspension, and soil moisture was determined gravimetrically (g/100 g). Soil organic matter content (% OM) was determined by loss on ignition (LOI) analysis. Soil texture was determined using a Bouyoucos densimeter after shaking the soil vigorously with NaOH 1 M as dispersant. Soil CaCO_3_ was determined a by Scheibler's method. Phosphate (P) was determined as the amount extracted from a soil after addition of water at a shaking ratio of 1 : 60, a procedure typically used to determine the soil fertility status of arable fields in The Netherlands. Cr, Cu, Hg, As, Ni, Cd, Pb, and Zn were extracted by Mehlich 1 and determined by atomic absorption spectrometry.

### 2.2. DNA Extraction, Amplification, Labelling, and Hybridization

DNA extractions were performed separately on each of the five replicates per field using the MoBio Power Soil Extraction kit (MoBio, Carlsbad, CA, USA) with bead-beating (Restch MM301, Retsch GmbH, Germany) at 5.5 m s^−1^ for 10 min. Total DNA concentration was quantified on a ND-1000 spectrophotometer (Nanodrop Technology, Wilmington, DE, USA).

The DNAs from replicates of each sample were pooled. Because the amount of DNA extracted was in most cases insufficient for direct labeling and hybridization, 30 ng of DNA per sample was amplified by whole community rolling cycle amplification (WCRCA) using a TempliPhi kit (GE Healthcare, Piscataway, NJ). We used the GeoChip functional microarray platform, which contains more than 24,000 oligonucleotide (50-mer) probes targeting targeted approximately 10,000 genes involved in nitrogen, carbon, sulfur and phosphorus transformations and cycling, metal reduction and resistance and organic xenobiotic degradation [18]. The number of technical replicates for microarray hybridization was four for samples of fields 1F, 4F, 10F, 13F, 16F, 19F, and 25F and three for field 8F. The amplified DNAs were labelled with a Cy5 fluorescent dye, purified, and then hybridized to the GeoChip functional array (FGA II) [18] in a Tecan hybridization station (Durham, NC) at 42°C for 10 hours. Arrays were washed, dried, and scanned using a ScanArray 5000. The signal intensity for each probe was determined digitally by Imagene software (Biodiscovery Inc., Los Angeles, CA).

### 2.3. Microarray Data Processing and Analysis

The data processing was according to He et al. [18]. Each array was cleaned by deleting flag 1.3 and SNR < 2 and normalized by mean of all spots in the same slide. The final table of all intensities of all samples were obtained by removing the outline spots more than 2 sigma, the maximal ratio between spots was 3 if certain genes had only 2 spots in the array, the spots with final spot numbers was 0.51 times less than the original number and the minimal spots number for a gene was 2.

### 2.4. Statistical Analysis

Pearson correlations were calculated between soil factors (total C, total N, C : N ratio, organic matter, soil pH, CaCO_3_, Cr, Cu, P, Zn, soil texture, and soil moisture) using the “multtest” package in R (version 2.6.0, The R Foundation for Statistical Computing). *P* values were corrected for multiple testing, using the false discovery rate controlling procedure [19]. The probe intensities and soil physicochemical factors were used for multivariate regression tree (MRT) analysis by using the “mvpart” package in R, and the distance matrix was based on Bray-Curtis built by the function “gdist.”

Microbial community functional structure was related to soil factors using canonical correspondence analyses (CCA) in Canoco 4.5 for Windows [20]. Probe intensities were used as “species” data, while soil data was included in the analysis as “environmental” variables. Variables having the most significant influence on the microbial community structure were chosen by forward selection with a *P* < 0.01 baseline. The variables selected this way were then included in a model whose significance was tested with 999 permutations. Gene functional category and land fields were added as extra variables but not involved in the calculations.

## 3. Results and Discussion

### 3.1. Soil Properties

In general, the pasture field 19F was very different from the other fields with highest cadmium, clay, chromium, copper, mercury, nickel, organic matter, silt, total carbon, total nitrogen, and zinc contents (Table 1). The pine forest 4F had higher C : N ratio, sand, and total C contents and lower cadmium, clay, chromium, pH, phosphate, and zinc contents than the other sites (Table 1). The highest percentage of CaCO_3_ was found in organic arable field 13F. The soil moisture at the time of sampling varied among fields. The moisture contents of 19F and 25F fields were the highest, while moisture contents of 10F and 1F fields were the lowest (Table 1).

The correlation analyses showed significant correlation between several soil factors. Soil pH was negatively correlated with C : N ratio. C : N ratio was negatively correlated with silt and chromium and positively correlated with sand. Total N was positively correlated with total C, organic matter, clay, chromium, and zinc and negatively correlated with sand. Chromium and zinc were positively correlated (Table S1).

### 3.2. Microarray Analyses

A total of 1405 genes across all samples were detected. The pasture field 1F had the lowest numbers of genes (146) detected by the GeoChip followed by conventional arable field 8F (173 genes) and natural grassland field 25F (Table 2). The organic arable field 13F had the highest number of genes detected (956). The pasture field 1F with lower functional gene number had high overlap with communities of natural grassland 25F (27.6%) and organic arable field 10F (25.9%) (Table 2). The shared genes between two or more field-soils varied from 12.2 to 37.6%, and the numbers of unique genes identified in only one soil were relative very small, varying from 2.7 to 19.7%, except for organic arable field 13F (33.4%) (Table 2). Although there were large differences in the number of genes detected in each field, fields with similar soil characteristics, such as high total nitrogen in 1F, 25F, and 10F and low phosphate content (Figure 2) in 13F and 19F showed the most overlap in detected genes.

**Figure 1:**
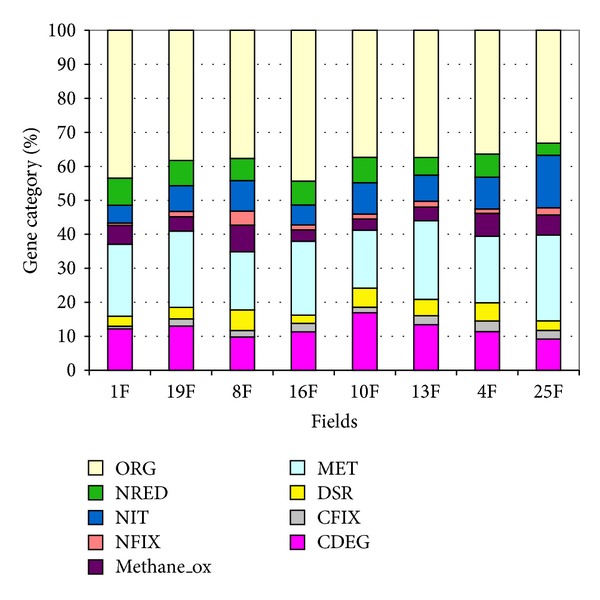
Percentage of gene categories given in the GeoChip present in soils from different land uses: pasture (1F, 19F), conventional arable field (8F, 16F), organic arable field (10F, 13F), forest (4F), and natural grassland (25F). CDEG: carbon degradation; CFIX: carbon fixation; DSR: dissimilatory sulfate reductase; MET: metal reductase; Methane_ox: methane oxidation NFIX: nitrogen fixation; NIT: nitrification; NRED: nitrogen reductase; ORG: organic remediation.

In general, there were differences in microbial community functional structure among the study sites (Figure 1). The percentage of genes related to carbon degradation (17%) was highest in organic arable fields (10F) and lowest in natural grassland 25F. This low representation of carbon degradation genes in field 25F is somewhat expected, as this particular field represents a typical Dutch “Blauwgrasland” soil type, which is often inundated and therefore exposed to anaerobic conditions. Indeed, soil water content impacts oxygen diffusion what higher moisture content leads to decrease in organic matter decomposition due to low oxygen supply [21]. Genes detected for carbon fixation were generally similar across the soils examined, except for 10F and 1F fields, which both had a lower percentage of genes from this category. Interestingly, these two fields had the lowest soil moisture contents. Dissimilatory sulfate reductase genes were highest (5-6%) in soils from filed 4F, 8F, and 10F. Pine forest field 4F and conventional arable field 8F had higher percentages (6-7%) of genes of methane oxidation. The conventional arable field 8F had highest number of genes of nitrogen fixation (4%), while fields 16F and 1F had highest gene numbers for organic remediation (44%) (Figure 1).

**Figure 2:**
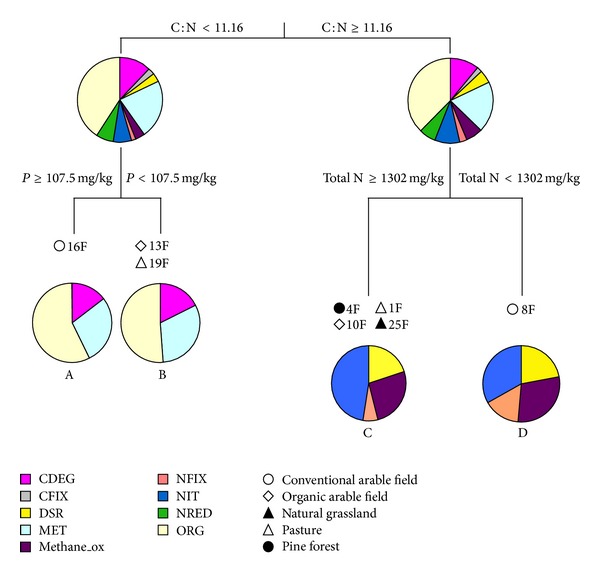
Multivariate regression tree (MRT) analysis of soil physicochemical properties and GeoChips intensities given by samples of fields 1F, 4F, 8F, 10F, 13F, 16F, 19F, and 25F. A, B, C, and D are clusters.

In a multivariate regression tree (MRT) analysis, combining the measured soil factors (pH, total N, total C, C : N ratio, organic matter, phosphate, clay, silt, sand, CaCO_3_, Cd, Cr, Cu, Ni, Pb, Zn, As, and Hg) and the intensities of the 1405 genes detected by the GeoChip, the main factor that explained the microbial community functional structure differences in the eight fields was C : N ratio (Figure 2). C : N ratio differentiated the fields into two main groups: (a) fields 16F (conventional arable field), 19F (pasture), and 13F (organic arable field) with C : N ratio lower than 11.15 and (b) fields 10F (organic arable field), 4F (pine forest), 25F (natural grassland), 1F (pasture), and 8F (conventional arable field) with C : N ratio higher than 11.15. The clustering of the three fields 13F, 16F, and 19F with C : N ratio lower than 11.16 also corresponds to less sand in the soil texture and higher soil pH as compared to the other fields, as C : N was significantly positively correlated with sand and soil pH.

The main differences between the two clusters A/B (fields 16F, 13F, and 19F) and C/D (fields 1F, 4F, 10F, 25F, and 8F) were that fields with C : N ratios lower than 11 had more genes for carbon degradation (CDEG), carbon fixation (CFIX), metal reductase (MET), and organic remediation (ORG) categories than the fields with C : N ratio higher than 11. C : N ratio is known to have a large influence on decomposition rates [22]. Fields 16F, 13F, and 19F had the lowest C : N ratios. It is expected that soils with lower C : N ratios will contain more easily decomposable organic matters as compared to soils with high C : N ratios [23]. Our results are consistent with this expectation, with higher numbers of carbon degradation genes in those sites with lowest C : N ratios. In addition, CO_2_ is released into the environment during the organic material decomposition, and this may explain the high numbers of microbial carbon fixation genes [24] in fields 16F, 13F, and 19F. On the other hand, fields with a C : N ratio higher than 11 showed more genes from categories dissimilatory sulfate reductase (DSR), methane oxidation, nitrification (NIT), and nitrogen fixation (NFIX) than fields with C : N lower than 11 (Figure 2).

**Figure 3:**
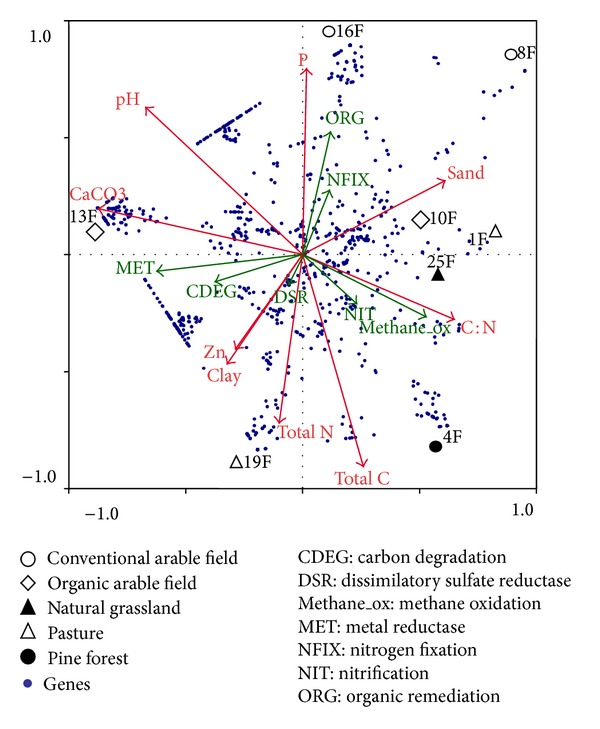
Canonical correspondence analysis (CCA) triplot of soil physicochemical properties (red arrows), GeoChips intensities (dots) with functional categories (green arrows), and eight fields with different land uses.

Among fields with C : N ratio lower than 11, field 16F had the highest number of genes of the organic remediation (ORG) category. This may be related to the high amount of phosphate in this soil—that is, greater than 107 mg/kg soil as calculated in MRT analysis and illustrated in the CCA plot (Figure 3). On the other hand, the high percentage of genes for CDEG and MET in fields 13F and 19F appeared to be explained by lower phosphate concentrations (less than 107 mg/kg; Figure 2). The higher phosphate contents in fields 13F, 16F, 19F and 16F may be related to the high clay content of these soils, as it is well known that phosphate availability strongly depends on clay quantity and quality [25].

The genes detected within the ORG category in 16F are related to a range of degradation activities (Table S2). In fact, phosphate has been shown to be an important nutrient factor required by bacterial biofilter for maximum methane elimination [26]. The field 16F is a conventional arable field that has been intensively cultivated with different crops. It has therefore been subjected to high inputs of inorganic fertilizers such as nitrogen, phosphate, and potassium. Phosphate was negatively correlated with total C and total C was positively correlated with organic matter content. In other words, the fields 13F and 19F had higher total carbon, higher organic matter and lower phosphate than field 16F, and these properties seem to be favourable for genes involved in carbon degradation (CDEG) and metal reductase (MET) activities (Figures 2 and 3). The CDEG genes overrepresented in samples 13F and 16F were related to bacterial and fungal cellulases; bacterial and fungal chitinases; fungal laccases; and bacterial, fungal, and oomycete polygalacturonases (Table S3). The MET genes overrepresented in these same fields 13F and 19F were genes encoding reductases of aluminium, arsenic, cadmium, chromium, cobalt, copper, cytochrome, lead, mercury, nickel, tellurium, and vanadium (Table S3), and some soil chemicals, that is, cadmium, chromium, copper, mercury, and nickel, were indeed highest in the pasture field 19F (Table 1).

Fields with a C : N ratio higher than 11 (1F, 4F, 10F, and 25F) had relatively high percentages of genes of nitrification (NIT), and high levels of total nitrogen (i.e., >1302 mg/kg) appeared to explain this result. The field 8F had the highest numbers of genes from categories for dissimilatory sulfate reductase, methane oxidation, and nitrogen fixation, and this pattern was explained by total N levels lower than 1302 mg/kg soil (Figure 2). The higher percentage of nitrogen fixation genes in field 8F than fields 1F, 4F, 10F, and 25F can be explained by the presence of nitrogen-fixing bacteria in this particular soil, probably related to the growth of legumes (beans) at this site. The nitrification-related genes abundant in fields 1F, 4F, 10F, and 25F were ureases,* amo*A,* pmo*A, and* ghd*. The DSR genes abundant in 8F were* dsr*A and* dsr*B; the methanol-oxidation category was dominated by* pmo*A and* mmo*A from methanotrophs and nitrogen fixation genes categorized as* nif*H from nitrogen-fixing bacteria (Table S4).

The nitrification genes found in fields 1F, 4F, 10F, and 25F were similar to uncultured ammonia-oxidizing *β* *-Proteobacteria amo*A genes. Ureases (E.C. 3.5.1.5) are complex metalloenzymes that catalyze the hydrolysis of urea to ammonia and carbon dioxide. This enzyme allows many soil bacteria to use urea as nitrogen source, and we detected several ureases in nitrogen-fixing bacteria such as* Mesorhizobium loti*,* Bradyrhizobium japonicus*,* Rhodopseudomonas palustris*,* Rhodobacter sphaeroides*,* Rhodobacter* (*α* *-Proteobacteria*),* Chromobacterium violaceum* (*β* *-Proteobacteria*), and* Nostoc* (*Cyanobacteria*) (Table S4). Urease is also an important virulence factor that improves survival of pathogenic bacteria in harsh conditions within the host and causes direct damage to the host due to ammonium, CO_2_, or alkali production (for reviews see [27, 28]), and in our study we found ureases similar to those of plant pathogens such* Pseudomonas syringae*,* Klebsiella aerogenes*, (*γ*-*Proteobacteria*),* Mycobacterium* (*Actinobacteria*), and* Brucella melitensis* (*α*-*Proteobacteria*) (Table S4).

Fields 4F and 25F had distinct soil properties or conditions that would be expected to impact numerous microbial processes. However, we found no evidence for a sharp distinction in the functional gene repertoires of these communities. Field 4F was extremely acidic which distinguishes it from other natural and cultivated soils. In fact, in a previous study on microbial community composition across 26 fields under different land uses including the eight fields here studied, Kuramae et al. [17] found that field 4F had the most distinct microbial community composition. This difference was linked to low pH and high C : N ratio. However, in the present study, 4F and 25F have similar microbial function profiles, despite the differences in soil pH, C : N ratio, and moisture. Thus, it appears to be that soil microbial composition is more sensitive to changes in soil pH than the functional capability of the community. This may be due to the function redundancy present in soil communities. In addition, the soil factors that drive microbial composition are not the same as those driving microbial potential function structures.

## 4. Conclusion

The soil-borne microbial functional structure in the different fields in The Netherlands did not group the sites accordingly to land management. Although the number of fields examined here was limited to eight, the breadth of our study was sufficient to assess the differences in microbial functional genes in different systems of soil management, and specific soil factors could be identified that explained the differences observed in functional gene composition of the different soils examined.

## Supplementary Material

Supplementary material is regarding to soil physicochemical factors, genes of organic remediation, carbon degradation, metal reductase and nitrogen fixation categories overrepresented in diffrent fields.

## Figures and Tables

**Table 1:** Soil physical and chemical variables measured in eight samples with different land uses.

Field	Land use	Vegetation	pH	Moisture	Organic matter	Clay	Silt	Sand	CaCO_3_	P	Total C	Total N	C : N ratio	Cr	Cu	Hg	As	Ni	Cd	Pb	Zn
				%						mg/L	g/100 g	mg/kg	mg/kg							
8F	Conventional arable field	Beans	5.8	17.15	3.5	0.6	10.9	88.4	0	69	2.1	1267	16.6	7.6	11	0.03	1.4	0	0.20	15	28
16F	Conventional arable field	Crops	7.3	15.57	1.7	11.6	27.2	61.2	0.2	146	1.1	1217	9.00	19	0	0.04	7.5	6.7	0.11	16	29
4F	Forest	Pine	3.7	16.91	6.4	0.3	4.3	95.4	0	24	3.8	1703	22.3	0	0	0.04	1.8	0	0.10	15	0
25F	Natural	Grassland	5.5	24.93	5.3	1.8	6.4	91.9	0	3	2.9	2029	14.3	7.2	6.2	0.06	3.4	3.5	0.24	9.2	12
10F	Organic arable field	Maize	5.8	13.14	3.6	1.8	10.2	88	0	121	2.0	1338	14.9	7.2	5.3	0.03	2.5	0	0.18	17	25
13F	Organic arable field	Crops	7.4	18.37	2.4	8.2	39.8	52	3.8	69	1.4	1440	9.7	26	8.2	0.04	13	10	0.20	15	40
1F	Pasture	Grassland	6.2	13.77	5.8	4.1	16.1	79.7	0.2	84	2.7	2142	12.6	5.5	21	0.05	26	0	0.32	37	43
19F	Pasture	Grassland	6.0	29.14	9.4	36.7	51.1	12.1	0.2	32	4.8	5489	8.7	52	27	0.08	14	33	0.49	32	130

**Table 2:** Total number and percentage of functional genes unique and overlapping in samples of different land uses.

% of genes	25F	19F	16F	13F	10F	8F	4F	1F
25F	*13 (6.9) *	132 (19.7)	136 (23.8)	145 (14.5)	119 (31.4)	61 (20.4)	123 (22.1)	72 (27.6)
19F		*113 (18.4) *	300 (36.0)	429 (37.6)	214 (30.1)	107 (15.7)	264 (31.3)	109 (16.7)
16F			*72 (13.8) *	381 (34.8)	206 (33.0)	110 (18.9)	251 (33.0)	116 (21.1)
13F				*319 (33.4) *	254 (25.1)	133 (13.3)	328 (29.3)	120 (12.2)
10F					*16 (5.1) *	100 (26.0)	186 (30.1)	94 (25.9)
8F						*16 (9.2) *	96 (16.8)	59 (22.7)
4F							*97 (19.7) *	120 (23.1)
1F								*4 (2.7) *

Total number of genes	187	614	520	956	311	173	493	146

Italic numbers represent unique genes in each soil sample.

The remaining numbers represent the numbers of genes and their percentage (in parenthesis) overlapping between two samples.
