# Supplementary material for: Soil-Borne Microbial Functional Structure across Different Land Uses
Source: ScientificWorldJournal. 2014 Aug 10;2014:216071. doi: 10.1155/2014/216071 (PMC4142738; doi:10.1155/2014/216071)
Supplement: Supplementary file 1 — Supplementary material is regarding to soil physicochemical factors, genes of organic remediation, carbon degradation, metal reductase and nitrogen fixation categories overrepresented in diffrent fields. [file 216071.f1.docx]

**Soil-borne microbial functional structure in different land use**

Eiko E. Kuramae^1*^, Jizhong Zhou^2^, George A. Kowalchuk^3^ and Johannes A. van Veen^1,4^

^1^Department of Microbial Ecology, Netherlands Institute of Ecology (NIOO-KNAW), Wageningen, The Netherlands.

^2^Institute for Environmental Genomics, University of Oklahoma, Norman, Oklahoma, USA.

^3^ Department of Biology, University of Utrecht, Utrecht, The Netherlands.

^4^Institute of Biology, Leiden University, Leiden, The Netherlands.

*For correspondence. E-mail [e.kuramae@nioo.knaw.nl](mailto:e.kuramae@nioo.knaw.nl)

Running title: Microbial functional structure in land use change

**Supplementary Material**

Table S1. Pearson correlation between soil physicochemical factors of eight different fields. Correlation values are given in the lower triangle of the matrix, and the two-tailed probabilities that the columns are uncorrelated are given in the upper.

|  | Phosphate | pH | Total N | Total C | C:N | OM | Clay | Silt | Sand | CaCO3 | Cr | Cu | Zn |
| --- | --- | --- | --- | --- | --- | --- | --- | --- | --- | --- | --- | --- | --- |
| Phosphate | 0 | 0,129 | 0,284 | **0,047** | 0,389 | 0,074 | 0,817 | 0,858 | 0,993 | 0,945 | 0,865 | 0,572 | 0,824 |
| pH | 0,583 | 0 | 0,869 | 0,093 | **0,003** | 0,178 | 0,499 | 0,112 | 0,223 | 0,163 | 0,272 | 0,709 | 0,463 |
| Total N | -0,433 | -0,070 | 0 | **0,015** | 0,366 | **0,006** | **0,003** | 0,089 | **0,026** | 0,732 | **0,019** | 0,022 | **0,002** |
| Total C | -0,713 | -0,632 | 0,810 | 0 | 0,586 | **0** | 0,200 | 0,737 | 0,464 | 0,344 | 0,413 | 0,169 | 0,200 |
| C:N | -0,354 | -0,887 | -0,371 | 0,229 | 0 | 0,793 | 0,082 | **0,015** | **0,027** | 0,339 | **0,037** | 0,284 | 0,087 |
| OM | -0,661 | -0,529 | 0,858 | 0,984 | 0,111 | 0 | 0,153 | 0,625 | 0,376 | 0,370 | 0,347 | 0,075 | 0,125 |
| Clay | -0,098 | 0,282 | 0,891 | 0,506 | -0,649 | 0,555 | 0 | **0,005** | **0** | 0,903 | **0** | 0,084 | **0** |
| Silt | 0,076 | 0,605 | 0,638 | 0,142 | -0,809 | 0,206 | 0,873 | 0 | **0** | 0,205 | **0** | 0,164 | **0,008** |
| Sand | -0,004 | -0,485 | -0,768 | -0,304 | 0,766 | -0,364 | -0,956 | -0,978 | 0 | 0,434 | **0** | 0,111 | **0,001** |
| CaCO3 | 0,029 | 0,544 | -0,145 | -0,386 | -0,390 | -0,368 | 0,052 | 0,502 | -0,324 | 0 | 0,481 | 0,947 | 0,879 |
| Cr | -0,072 | 0,443 | 0,791 | 0,338 | -0,736 | 0,384 | 0,958 | 0,952 | -0,985 | 0,293 | 0 | 0,122 | **0,001** |
| Cu | -0,237 | 0,158 | 0,783 | 0,538 | -0,433 | 0,660 | 0,645 | 0,543 | -0,606 | -0,028 | 0,592 | 0 | **0,010** |
| Zn | -0,094 | 0,304 | 0,899 | 0,507 | -0,641 | 0,589 | 0,946 | 0,844 | -0,916 | 0,065 | 0,917 | 0,837 | 0 |

Pearson correlation: bold black color=positive; bold red color=negative correlation

Table S2. Genes from organic remediation (ORG) category more abundant in sample 16F given in the GeoChips.

| **Gene short description** | **Organism** |
| --- | --- |
| 2,4-D | uncultured bacterium, *Burkholderia* sp. |
| Acetylene | *Agrobacterium tumefaciens, Mesorhizobium loti* |
| Acrylonitrile | uncultured bacterium |
| Aniline | *Acinetobacter* sp., *Mycobacterium bovis, Sinorhizobium meliloti* |
| Atrazine | *Yersinia pestis* |
| Benzoate-aerobic | *Acinetobacter calcoaceticus* |
| Benzoate-anaerobic | *Acinetobacter* sp., *Rhodopseudomonas palustris, Ustilago maydis* |
| Benzonitrile | *Rhodococcus rhodochrous, Rhodopseudomonas palustris, Xanthomonas campestris* |
| Biphenyl | *Novosphingobium aromaticivorans, Rhodococcus erythropolis, Sphingobium yanoikuyae* |
| Catechol | *Pseudomonas chlororaphis, Ralstonia pickettii, Rhodopseudomonas palustris*, uncultured bacterium, |
| Catechol-meta derivative | *Comamonas* sp., *Mycobacterium bovis*, *Pirellula* sp., *Pseudomonas* sp., *P. putida*, *Rhodococcus opacus* |
| Cyanuric acid | *Leuconostoc mesenteroides, Pseudomonas aeruginosa, Rhodobacter sphaeroides* |
| Dibenzothiophene | *Bacillus subtilis* |
| Dichloromethane | *Methylophilus* sp. |
| DMSO | *Actinobacillus pleuropneumoniae* |
| Gentisate | *Sphingomonas* sp. |
| g-hexachlorocyclohexane | *Xylella fastidiosa* |
| Haloacid | *Xanthobacter autotrophicus* |
| Hydroxyacetophenone | *Sinorhizobium meliloti* |
| Limonene | *Rhodococcus erythropolis* |
| Naphthalene | *Novosphingobium aromaticivorans, Pseudomonas putida, Ralstonia* sp. |
| Nitrilotriacetate | *Agrobacterium tumefaciens,Sinorhizobium meliloti* |
| Nitrobenzoate | *Pseudomonas* sp. |
| Phenanthrene | *Alcaligenes faecalis, Nocardioides* sp. |
| Phenol-aerobic | *Xanthomonas axonopodis, Ralstonia* sp. |
| Phenoxybenzoate | *Pseudomonas pseudoalcaligenes* |
| Phenylpropionate | *Leuconostoc mesenteroides, Azotobacter vinelandii* |
| Phthalate | *Burkholderia cepacia, Mycobacterium vanbaalenii* |
| Protocatechuate | *Azotobacter vinelandii, Bradyrhizobium japonicum, Leuconostoc mesenteroides, Rhodobacter sphaeroides, Pseudomonas syringae,* |
| Salicylate | *Streptomyces* sp. |
| Thiocyanate | *Bradyrhizobium japonicum, Oligotropha carboxidovorans* |
| Vanillin | *Azotobacter chroococcum, Pseudomonas putida, Ralstonia solanacearum* |

Table S3. Genes of Carbon degradation (CDEG) and Metal reductase (MET) categories more abundant in fields F13 and F19 than filed F16.

| **Gene category** | **Gene short description** | **Organism** |
| --- | --- | --- |
| CDEG | Cellulase (Bacteria) | *Agrobacterium tumefaciens*, *Cellulomonas* *fimi*, *Chromobacterium violaceum*, *Clavibacter michiganensis*, *Leuconostoc mesenteroides*, *Mesorhizobium loti*, *Mycobacterium tuberculosis*, *Pectobacterium carotovorum, Reticulitermes speratus*, *Rhodopseudomonas palustris, Salmonella typhimurium*, *Streptomyces coelicolor*, *Xanthomonas* sp. |
|  | Cellulase (Fungi) | *Aspergillus aculeatus, Humicola grisea, Neurospora crassa, Piromyces* sp*.* |
|  | Chitinase (Bacteria) | *Bacillus circulans, Burkholderia fungorum, B. gladioli, Coniothyrium minitans, Mycobacterium bovis, Nocardiopsis prasina, Salinivibrio costicola, Serratia marcescens, Xanthomonas sp.* |
|  | Chitinase (Fungi) | *Ajellomyces capsulatus*, *Rhizopus niveus*, *Schizosaccharomyces pombe* |
|  | Laccase | *Gaeumannomyces graminis, Heterobasidion annosum, Lachnum spartinae, Piloderma byssinum, Pleurotus sajor-caju, Polyporus ciliatus, Trametes sp., T. versicolor, T. villosa,* uncultured basidiomycete |
|  | Mannanase | *Bacteroides thetaiotaomicron* |
|  | pgl | *Brucella suis, Mycobacterium leprae, Pseudomonas aeruginosa, Xanthomonas axonopodis* |
|  | Polygalacturonase (Bacteria) | *Erwinia chrysanthemi, Ralstonia solanacearum* |
|  | Polygalacturonase (Fungi/Oomycete) | *Kluyveromyces wickerhamii, Penicillium olsonii, P. griseoroseum, Phytophthora cinnamomi* |
| MET | Aluminum | *Listeria monocytogenes* |
|  | Arsenic | *Acidiphilium multivorum, Azotobacter vinelandii, Bacillus halodurans, Bordetella parapertussis, Desulfitobacterium hafniense, Desulfovibrio desulfuricans, Escherichia coli, , Klebsiella oxytoca, , Leuconostoc mesenteroides, Magnetospirillum magnetotacticum, Mycobacterium bovis, M. tuberculosis, Pseudomonas putida, Rhodococcus erythropolis, R. sphaeroides, Salmonella typhimurium, Serratia marcescens, Staphylococcus epidermidis* |
|  | Cadmium | *Alcaligenes* sp., *Schizosaccharomyces pombe* |
|  | Chromium | *Caulobacter crescentus, Chromobacterium violaceum, Leuconostoc mesenteroides, Magnetospirillum magnetotacticum, Mycobacterium bovis, Pseudomonas aeruginosa, Rhodobacter sphaeroides, Sinorhizobium meliloti* |
|  | Cobalt, Zinc, Cadmium | *Xanthomonas campestris* |
|  | Copper | *Bradyrhizobium japonicum*, *Leuconostoc mesenteroides, Pseudomonas fluorescens, Xanthomonas campestris* |
|  | Cytochrome | *Desulfovibrio vulgaris, Geobacter sulfurreducens, Rhodopseudomonas palustris, Shewanella oneidensis* |
|  | Lead | *Pirellula* sp. |
|  | Mercury | *Acinetobacter* sp., *Acidithiobacillus ferrooxidans, Citrobacter koseri, Mesorhizobium loti, Pseudomonas sp., P. stutzeri, Salmonella typhimurium,*  *Shewanella oneidensis, Streptomyces coelicolor, Xanthomonas campestris* |
|  | Nickel | *Escherichia coli, Pseudomonas syringae, Ruegeria* sp. |
|  | Nickel, Cobalt, Cadmium | *Mesorhizobium loti* |
|  | Tellurium | *Azotobacter vinelandii, Corynebacterium glutamicum, Escherichia coli, Leuconostoc mesenteroides, Pseudomonas syringae, Rhodobacter sphaeroides, Shigella flexneri, Streptomyces coelicolor* |
|  | Vanadium | *Pseudomonas* *aeruginosa* |
|  | Zinc | *Staphylococcus aureus* |
|  | Zinc, Cadmium | *Schizosaccharomyces pombe* |
|  | Cadmium, Zinc, Cobalt | *Zymomonas mobilis* |

Table S4. Genes of nitrification (NIT) category more abundant in fields F1, F4, F10 and F25 than field F8.

| **Gene short description** | **Organism** |
| --- | --- |
| amoA | uncultured bacterium, uncultured ammonia-oxidizing *beta proteobacterium*, unidentified bacterium |
| amoA/pmoA | uncultured bacterium |
| gdh | *Enterococcus faecalis, Trypanosoma brucei* |
| urease | *Bacillus* sp*., Bradyrhizobium japonicum, Brucella* sp*., Chromobacterium violaceum, Deinococcus radiodurans, Klebsiella aerogenes, Lactobacillus fermentum, Mesorhizobium loti, Mycobacterium tuberculosis, Nostoc* sp.*, Pseudomonas aeruginosa, P. syringae, Rhodobacter capsulatus, R. sphaeroides Rhodopseudomonas palustris, Streptomyces avermitilis, Wautersia eutropha* |
